# Nanoarchitectured Nb_2_O_5_ hollow, Nb_2_O_5_@carbon and NbO_2_@carbon Core-Shell Microspheres for Ultrahigh-Rate Intercalation Pseudocapacitors

**DOI:** 10.1038/srep21177

**Published:** 2016-02-16

**Authors:** Lingping Kong, Chuanfang Zhang, Jitong Wang, Wenming Qiao, Licheng Ling, Donghui Long

**Affiliations:** 1 State Key Laboratory of Chemical Engineering, East China University of Science and Technology, Shanghai 200237, China; 2 Key Laboratory of Specially Functional Polymeric Materials and Related Technology, East China University of Science and Technology, Shanghai 200237, China

## Abstract

Li-ion intercalation materials with extremely high rate capability will blur the distinction between batteries and supercapacitors. We construct a series of nanoarchitectured intercalation materials including orthorhombic (*o*-) Nb_2_O_5_ hollow microspheres, *o*-Nb_2_O_5_@carbon core-shell microspheres and tetragonal (*t*-) NbO_2_@carbon core-shell microspheres, through a one-pot hydrothermal method with different post-treatments. These nanoarchitectured materials consist of small nanocrystals with highly exposed active surface, and all of them demonstrate good Li^+^ intercalation pseudocapacitive properties. In particular, *o*-Nb_2_O_5_ hollow microspheres can deliver the specific capacitance of 488.3 F g^−1^, and good rate performance of 126.7 F g^−1^ at 50 A g^−1^. The *o*-Nb_2_O_5_@carbon core-shell microspheres show enhanced specific capacitance of 502.2 F g^−1^ and much improved rate performance (213.4 F g^−1^ at 50 A g^−1^). Furthermore, we demonstrate for the first time, *t*-NbO_2_ exhibits much higher rate capability than *o*-Nb_2_O_5_. For discharging time as fast as 5.9 s (50 A g^−1^), it still exhibits a very high specific capacitance of 245.8 F g^−1^, which is 65.2% retention of the initial capacitance (377.0 F g^−1^ at 1 A g^−1^). The unprecedented rate capability is an intrinsic feature of *t*-NbO_2_, which may be due to the conductive lithiated compounds.

Interests in the development of electrochemical supercapacitors for high power applications have greatly intensified in recent years^1^,^2^. There are many electrode materials that are under close scrutiny, such as porous carbon materials, transition metal oxides and electronically conducting polymers^3^,^4^,^5^. The capacitance in the case of porous carbon materials is mainly due to the electric double layer formation at the electrode-electrolyte interface, while for the transition metal oxides and the conducting polymers, it is due to fast faradaic reaction^6^,^7^,^8^,^9^,^10^. Generally, three types of faradaic reaction have been recognized as pseudocapacitive processes. They are reversible surface adsorption (for example, adsorption of hydrogen on the surface of platinum)^11^, redox reactions of transition metal oxides (e.g., RuO_2_, MnO_2_, NiCo_2_O_4_)^12^,^13^,^14^ and electrochemical doping-dedoping in conductive polymers (e.g., polyaniline, polypyrrole)^15^,^16^. The first two processes are primarily surface or near-surface reversible redox reactions, while the third process is more of a bulk process^17^.

Recently, it was firstly recognized by Bruce Dunn *et al.* that capacitive-type lithium ions insertion/extraction reaction could occur not at the surface but in the bulk orthorhombic Nb_2_O_5_ in non-aqueous Li^+^ electrolyte^18^. The intercalation pseudo-capacitive behavior was highly dependent upon the presence of a crystalline structure. Amorphous and pseudo-hexagonal Nb_2_O_5_ exhibited lower specific capacitance values than *o*-Nb_2_O_5_^19^. This was due to the unique orthorhombic phase structure, which could provide fast two dimensional transport paths for Li^+^ between atomic layers. Thus, the kinetics was not diffusion-limited so that the overall electrochemical behavior was capacitive. This material represents capacities typical of battery materials but at rates closer to those of supercapacitors, which may open the door to a new energy storage concept that materials can possess battery and capacitor properties simultaneously. So far, only a few crystalline host materials have been identified to exhibit intercalation pseudocapacitive behavior, such as cation-intercalation type titanium carbide^20^ and oxygen-intercalation type LaMnO_3_ perovskite^21^ in aqueous electrolyte, and Li^+^-intercalation type Nb_2_O_5_ in organic electrolyte^8^. It would be of great importance to explore new analogous materials with a crystalline network for high-rate energy storage.

Similar to most pseudocapacitive metal oxides, Nb_2_O_5_ is an electronic semi-conductor with a bulk electrical conductivity of ~3.4 × 10^−6^ S cm^−1^ at 300 K^22^,^23^. When Nb_2_O_5_ nanocrystals are fabricated into a relatively thick practical electrode, the electrochemical utilization and high-rate property would be limited due to an increase of the ohmic polarization and ion diffusion constraints. It is known that as the dimensions of metal oxide crystals are reduced, their pseudocapacitive responses increase markedly, due to a high density of active surface and short pathways^24^,^25^,^26^. In addition, nanostructured composites with high conductivity and more exposed electroactive sites could further promote the pseudocapacitive properties. For example, Wang *et al.* prepared the CNT-Nb_2_O_5_ composites *via* a physical mixing, which could enable fast electron transport, and thus improve the rate capability^27^. Zhang *et al.* reported that the hydrothermal growth of Nb_2_O_5_ nanoparticles on carbide-derived carbons and consequent CO_2_ heat treatment could result in an increased gravimetric capacitance (157 C g^−1^) at a charge-discharge time of 3 min^28^. Our recently work also demonstrated that the synergistic effects between graphene and Nb_2_O_5_ nanoparticles, including minimizing the particle size, preventing particles from agglomerating, and facilitating electron and proton conduction, could give the composites very high capacitance and excellent rate capability^29^. All these results suggest that nanostructured technology is a superior strategy to boost the electrochemical capacitive performance of the intercalation electrode. Thus, developing synthetic procedure that yields optimized nanostructure composites would intrigue considerable interest.

In this work, we successfully constructed a series of nanoarchitectured materials including *o*-Nb_2_O_5_ hollow microspheres, *o*-Nb_2_O_5_@carbon core-shell microspheres and *t-*NbO_2_@carbon core-shell microspheres, through a one-pot hydrothermal method with different post-treatments. This synthetic procedure was straightforward and inexpensive, and consequently can be readily adopted to produce larger quantities of nanostructured microspheres. All these nanoarchitectured materials consisted of small nanocrystals with highly exposed active surface and shorter ion transport path. It was confirmed that the hollow *o*-Nb_2_O_5_ microspheres should be favored for intercalation electrodes, as they could provide hollow centers, which increases the surface-to-bulk ratio thus increasing the contact area between the active material and electrolyte. Compared with *o*-Nb_2_O_5_ hollow microspheres, *o*-Nb_2_O_5_@carbon core-shell microspheres showed enhanced specific capacitance and improved rate performance. These results confirmed that as two functional materials were constructed in a programmed way, possible synergetic efforts and better electrochemical properties could be achieved. More importantly, we found for the first time, that *t*-NbO_2_ nanocrystals exhibited much better charge storage kinetics than the *o*-Nb_2_O_5_. It exhibited considerable specific capacitance at ultrahigh rates, with nearly 245.8 F g^−1^ being stored reversibly within 5.9 s (a current density 50 A g^−1^). This value is higher than what has been reported for the Nb_2_O_5_ and other metal oxides^30^,^31^,^32^. The ultrafast Li^+^ intercalation kinetics of *t*-NbO_2_ may open up exciting possibilities of producing improved intercalation electrodes for high-power supercapacitors.

## Results

### Material synthesis and characterization

**Figure 1:**
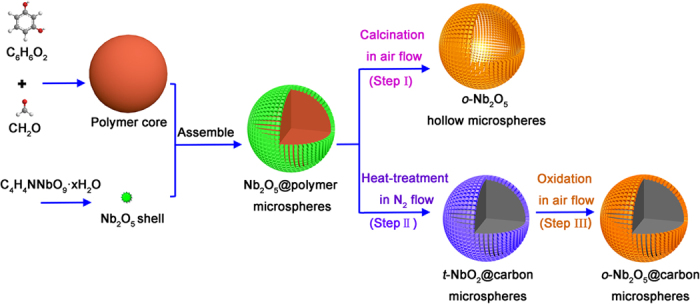
Schematics of the fabrication process: amorphous Nb_2_O_5_@polymer core-shell microspheres were obtained through hydrothermal method, followed with different post-treatments: (Step I) calcination in air flow at 600 ^o^C; (Step II) heat-treatment in N_2_ flow at 800 ^o^C; (Step III) a mild oxidation of *t*-NbO_2_@carbon microspheres in air flow at 300 ^o^C.

The synthesis and conversion strategy of *o*-Nb_2_O_5_ hollow microspheres, *o*-Nb_2_O_5_@carbon and *t*-NbO_2_@carbon core-shell microspheres is schematically illustrated in Fig. 1. Amorphous Nb_2_O_5_@polymer core-shell microspheres (Figure S1) are firstly prepared via a one-pot hydrothermal process, using resorcinol (R) and formaldehyde (F) as polymeric core precursor and ammonium niobate oxalate hydrate as Nb_2_O_5_ precursor. The formation of Nb_2_O_5_@polymer core-shell microspheres is accomplished by the “couple synthesis” approach, which should involve the fast formation of RF polymeric microspheres *in situ*, followed by the hetero-nucleation and growth of Nb_2_O_5_ nanoparticles on the RF polymeric microsphere surfaces. This process could be well verified by a hydrothermal time-dependent experiment (Figure S2).

**Figure 2:**
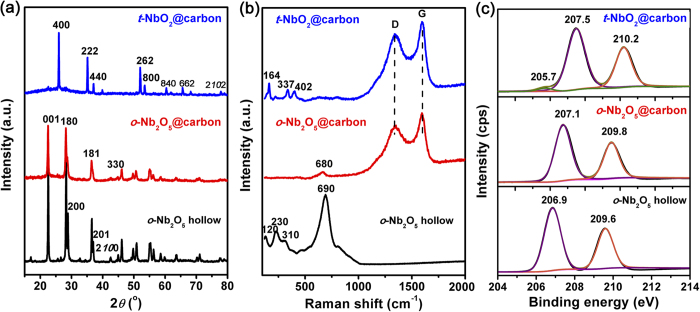
(**a**) XRD patterns, (**b**) Raman spectra and (**c**) high-resolution Nb3d XPS spectrum of *o*-Nb_2_O_5_ hollow microspheres, *t*-NbO_2_@carbon core-shell microspheres and *o*-Nb_2_O_5_@carbon core-shell microspheres.

The obtained Nb_2_O_5_@polymer core-shell microspheres could be converted into three kinds of nanoarchitectured materials through heat-treatment in air or N_2_ atmosphere. As illustrated in Fig. 1, *o*-Nb_2_O_5_ hollow microspheres are obtained by direct calcination of Nb_2_O_5_@polymer core-shell microspheres in air flow at 600 ^o^C (Step I). The *o*-Nb_2_O_5_ crystalline structure could be confirmed by XRD, Raman results and XPS analysis (Fig. 2). The hollow microspheres exhibit a high degree of crystallinity with an orthorhombic unit cell (JCPDS No. 30-0873) with diffraction peaks at 22.6^o^ (001), 28.3^o^ (180), 28.9^o^ (200), 36.5^o^ (181), 37.0^o^ (201), 42.5^o^ (2100), 45.0^o^ (330) for typical *o*-Nb_2_O_5_ crystals^19^,^33^. The specific Raman vibrational modes centered at 120 cm^−1^ (*v*_1_, the vibrations of octahedrons as a whole), 230 cm^−1^ and 310 cm^−1^ (*v*_2_ and *v*_3_, the vibrations of cations located inside the octahedrons and tetrahedrons, and 690 cm^−1^ (*v*_4_, the stretching Nb–О bonds), also confirm the *o*-Nb_2_O_5_ phase^34^,^35^. The high-resolution Nb3d XPS spectrum has peaks for Nb3d_5/2_ at 206.9 eV and Nb3d_3/2_ at 209.6 eV, in good agreement with the binding energies of Nb_2_O_5_^36^.

Direct carbonization of Nb_2_O_5_@polymer core-shell microspheres in N_2_ flow results the formation of *t*-NbO_2_@carbon core-shell microspheres (Step II). The tetragonal phase of NbO_2_ can be well revealed by its XRD pattern, which is indexed to JCPDS No. 43-1043 with diffraction peaks at 2θ of 26.0^o^ (400), 35.2^o^ (222), 37.1^o^ (440), 39.9^o^ (402), 52.1^o^ (262), 53.5^o^ (800), 60.4^o^ (840), 65.6^o^ (662), 68.3^o^ (404) and 78.0^o^ (2*10*2)^37^. The XRD pattern of *t*-NbO_2_@carbon also shows two small peaks at 22.6^o^ and 28.3^o^ corresponding to (001) and (180) planes of *o*-Nb_2_O_5_, respectively. The peaks in Raman spectrum are around 164 cm^−1^ (*v*_1_), 337 cm^−1^ (*v*_2_) and 402 cm^−1^ (*v*_3_), which are in consistent with the fingerprints of *t*-NbO_2_^38^,^39^. In addition, three peaks in Nb3d XPS spectrum are solved at 205.7 eV (Nb3d_5/2_, Nb^ + 4^), 207.5 eV (Nb3d_5/2_, Nb^+5^) and 210.2 eV (Nb3d_3/2_, Nb^+5^), in good agreement with the binding energies of *t*-NbO_2_^40^,^41^. The presence of Nb^+4^ species agrees with XRD and Raman indication, which suggests that amorphous Nb_2_O_5_ are mainly reduced to *t*-NbO_2_ instead of *o*-Nb_2_O_5_. This is possibly due to a reduction effect by carbon in N_2_ atmosphere during heat treatment process.

A mild oxidation of *t*-NbO_2_@carbon core-shell microspheres at 300 ^o^C in air flow could transfer *t*-NbO_2_ phase into *o*-Nb_2_O_5_ phase (Step III). The obtained *o*-Nb_2_O_5_@carbon core-shell microspheres show similar XRD structure with *o*-Nb_2_O_5_ hollow microspheres. From Raman spectrum, there is only a strong peak appeared at around 680 cm^−1^ (Nb-O-Nb bridging bond of distorted NbO_6_) that corresponds to a fingerprint of *o*-Nb_2_O_5_ phase^42^. XPS result indicates that the successful transition of *t*-NbO_2_ phase to *o*-Nb_2_O_5_ phase, as Nb3d_5/2_ (Nb^+4^) peak disappeared. The other two peaks in *o*-Nb_2_O_5_@carbon core-shell microspheres show little shifts to higher binding energy in contrast to that of *o*-Nb_2_O_5_ hollow microspheres, and these shifts might be caused by the incomplete oxidation of *t*-NbO_2_. It should be noted that the oxidation do not apparently alter the structure of carbon cores, as the intensities of D-Raman peak to G-Raman peak (*I*_D_/*I*_G_) are almost the same before and after oxidation. The weight contents of NbO_2_ and Nb_2_O_5_ in microspheres are 33.3 wt% and 34.0 wt% respectively, as determined by TG in air flow (Figure S3).

The porosity of these samples is shown in Figure S4 and Table S1. The *o*-Nb_2_O_5_ hollow microspheres have a relatively BET surface area of 26 m^2^/g. After compositing with carbon core, the BET surface areas of *t*-NbO_2_@carbon and *o*-Nb_2_O_5_@carbon core-shell microspheres increase to 473 m^2^/g and 456 m^2^/g, respectively. The increased surface areas are apparently due to the contribution of microporous carbon cores.

**Figure 3:**
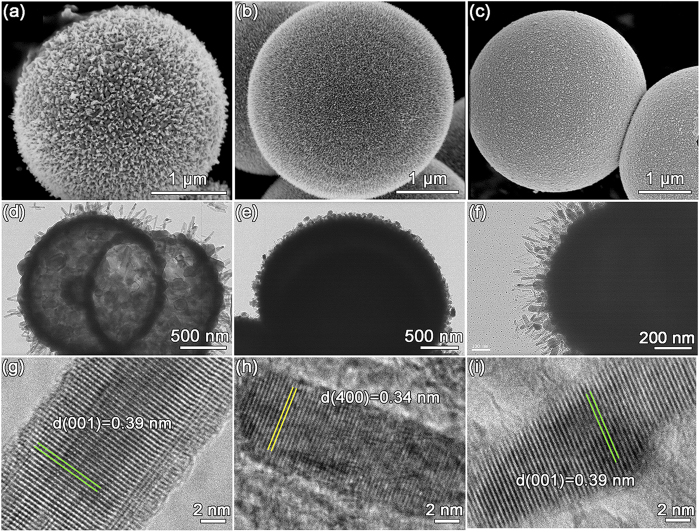
SEM, TEM and HR-TEM images of *o*-Nb_2_O_5_ hollow microspheres (**a,d,g**), *t*-NbO_2_@carbon core-shell microspheres (**b,e,h**) and *o*-Nb_2_O_5_@carbon core-shell microspheres (**c,f,i**).

The morphologies of the nanoarchitectured materials are observed by SEM and TEM. As shown in Fig. 3a–c, all the nanoarchitectured materials consist of spherical particles with a diameter of 2-3 μm. More SEM images are provided in Figure S5. Their surfaces show urchin-like shell assembled by numerous nanorods protruding radially from the center. These protruding nanorods offer relatively high contact area between active material and electrolyte, which should provide short and more efficient ion transport. The TEM images further reveal the detailed core-shell structure of these materials. In Fig. 3d, one can easily distinguish between the dark image in shell and the lighter region in core, indicating the hollow structure of *o*-Nb_2_O_5_ microspheres. The lattice fringes from *o*-Nb_2_O_5_ nanocrystals can be clearly observed in Fig. 3g. The distance between two adjacent lattice fringes has been found to be 0.39 nm which is within measurement error, consistent with (001) plane of orthorhombic Nb_2_O_5_. The TEM images show that *t*-NbO_2_@carbon and *o*-Nb_2_O_5_@carbon core-shell microspheres have quite similar morphological characteristics, with thick carbon core and thin urchin-like shell consisted of protruding nanorods. The difference in lattice could be observed in HR-TEM images, where *t*-NbO_2_ nanocrystals consist of (400) plane with spacing 0.34 nm while *o*-Nb_2_O_5_ nanocrystals includes (001) plane with spacing 0.39 nm.

### Electrochemical characterization

Orthorhombic Nb_2_O_5_ crystals have been identified as a pseudocapacitive material that exhibited intrinsic intercalation pseudocapacitance and did not limit to thin film electrode^8^,^18^,^19^. However, for a thick electrode containing insulating PVDF binder, the high-rate performance of Nb_2_O_5_ should be deteriorated due to the poor electron conductivity and limited ionic transport throughout the internal volume of thicker electrode layer. To emphasize the importance of nanostructured materials on electrode performance, a practical electrode with a thickness of ca. 50 μm was used. In preliminary experiments, we studied the electrochemical performance of pure carbon microspheres (Figure S6). We found that the electric double-layer capacitance of carbon microsphere was negligible compare to the pseudo-capacitance of Nb_2_O_5_. For facile comparison, the gravimetric current and capacitance are calculated based on the weight of active materials (Nb_2_O_5_ or NbO_2_).

**Figure 4:**
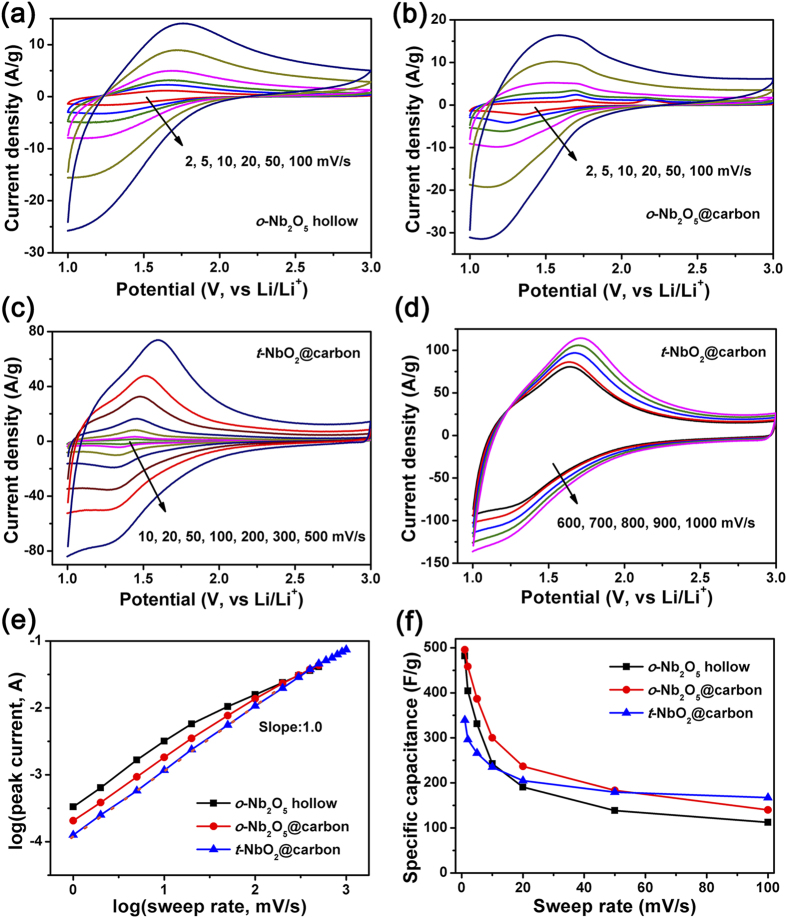
CV curves of *o*-Nb_2_O_5_ hollow microspheres (**a**), *o*-Nb_2_O_5_@carbon core-shell microspheres (**b**) and *t*-NbO_2_@carbon core-shell microspheres (**c,d**). The b-value from plot of log(*i*) versus log(*v*) for cathodic current peak (**e**). Specific capacitance versus sweep rate (**f**).

Cyclic voltammograms of *o*-Nb_2_O_5_ hollow microspheres and *o*-Nb_2_O_5_@carbon core-shell microspheres from 2 to 100 mV s^−1^ are compared in Fig. 4a,b. It is evident that both of them exhibit broad anodic and cathodic peak with small voltage separation in sweep rate range from 2 to 20 mV s^−1^. This characteristic is one of indicatives of pseudocapacitive behavior^18^. When the sweep rates are beyond 20 mV s^−1^, there is a noticeable peak shift due to the ohmic contribution. The specific capacitances are calculated by integrating the discharge portions of CV plots. As specific capacitance versus sweep rate plot presents in Fig. 4f, the specific capacitance and its retention of *o*-Nb_2_O_5_@carbon core-shell microspheres are significantly higher than these of *o*-Nb_2_O_5_ hollow microspheres at various sweep rates.

The kinetic characterization is studied by plotting log(*i*) versus log(*v*) for cathodic current peak with varied sweep rates, which can be used to distinguish the charge storage whether arises diffusion-controlled or capacitive processes. This relation is expressed as *i* = *av*^*b*^ with the value of *b* providing insights regarding the charge storage mechanism. Whereas a *b*-value of 0.5 indicates that the current is controlled by semi-infinite linear diffusion, and a value of 1 indicates that the current is capacitive process^18^,^43^. In sweep rate range of 1–20 mV s^−1^, the *b*-value of *o*-Nb_2_O_5_ hollow microspheres is very close to 1 but deviates severely at high rates (50–200 mV s^−1^), as shown in Fig. 4e. The hollow structures can provide a hollow center which increases the surface-to-bulk ratio. However, the limitation of active material resistance may deteriorate its rate capability at high rates. In contrast, *o*-Nb_2_O_5_@carbon core-shell microspheres has a *b*-value of 1 at a relatively wide range from 1 to 50 mV s^−1^, indicating the improved rate handling properties. The core-shell structure, in which conductive carbon microsphere is the core and *o*-Nb_2_O_5_ nanorods with short ion transport path is the shell, should be the reason why the electrochemical utilization and rate handling properties of *o*-Nb_2_O_5_ are improved.

There are three oxides of niobium: NbO, NbO_2_ and Nb_2_O_5_. While *o*-Nb_2_O_5_ has been reported as a pseudocapacitive material due to its unique crystalline structure, the charge storage in NbO_2_ has never been reported so far. As such, this work studies for the first time the charge storage in *t*-NbO_2_. The electrochemical properties of *t*-NbO_2_@carbon core-shell microspheres are firstly investigated by CV under the potential range of 1.0–3.0 V (vs. Li/Li^+^) with the sweep rate range from 1–1000 mV s^−1^. The *t*-NbO_2_ also exhibit a pair of well-defined cathodic and anodic peaks, which is similar to the electrochemical properties of *o*-Nb_2_O_5_. Kumagai *et al.* have revealed that the continuous variation in valence state from Nb^5+^ to Nb^4+^ takes place in Li^+^ intercalation reaction of Nb_2_O_5_^33^:


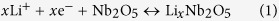


Similarly, the Li^+^ intercalation reaction of NbO_2_ could be proposed as:


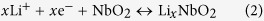


The redox couple of Nb^4+/3+^ should take place during Li^+^ intercalation reaction of NbO_2_. Thus, the reduction peaks at around 1.5 V result from Nb^4+^ to Nb^3+^, while the oxidation peaks at around 1.6 V is from Nb^3+^ to Nb^4+^. For clear comparison of Li^+^ intercalation potential of NbO_2_ and Nb_2_O_5_, the CV curves of *o*-Nb_2_O_5_ hollow, *o*-Nb_2_O_5_@carbon and *t*-NbO_2_@carbon at 1 mV s^−1^ are shown in Figure S7a. The Li^+^ insertion and extraction peaks of *t*-NbO_2_@carbon are at around 1.35 V and 1.42 V, respectively. While, *o*-Nb_2_O_5_ hollow sample shows two insertion peaks at 1.17 V and 1.41 V and one broad extraction peaks at 1.60 V. Apparently, *t*-NbO_2_ and *o*-Nb_2_O_5_ show the different Li^+^ intercalation potential, which could be also confirmed by the following electrochemical tests of commercial *t*-NbO_2_ and *o*-Nb_2_O_5_ powders in Figure S7b. The *t*-NbO_2_ and *o*-Nb_2_O_5_ provide different interstitial sites for Li^+^ result in different intercalation potential. While, the broad insertion/extraction peaks indicate that their intercalation sites exhibit the broad Li^+^ adsorption energy distribution.

The specific capacitance of *t*-NbO_2_@carbon is 235.1 F g^−1^ at a charging time of 200 s (10 mV s^−1^), lower than 242.7 F g^−1^ for *o*-Nb_2_O_5_ hollow and 300.1 F g^−1^ for *o*-Nb_2_O_5_@carbon. However, with charging time decreasing, the capacitance of *t*-NbO_2_ decreases slightly and over 47.9% of the capacity can be maintained within 2 s, much higher than these for *o*-Nb_2_O_5_. The kinetic information obtained from CV curves is shown in Fig. 4e. The *b*-value of *t*-NbO_2_ is close to 1 for cathodic peak currents in a wide sweep rate range of 1 to 500 mV s^−1^, further indicting that an ultrafast Li^+^ intercalation process with a capacitive behavior takes place in *t*-NbO_2_. The kinetic response suggests that *t*-NbO_2_ possesses much better charge storage kinetics than *o*-Nb_2_O_5_.

**Figure 5:**
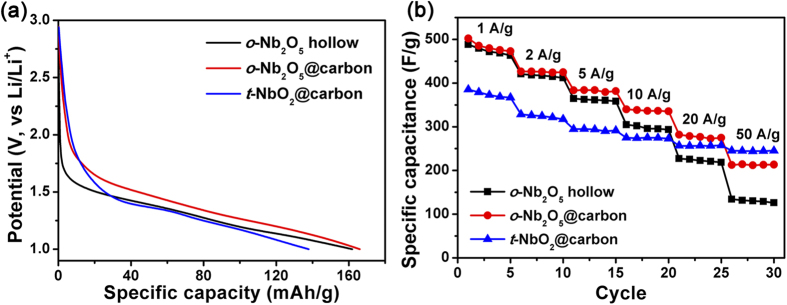
Galvanostatic charge-discharge curves (**a**) and rate capability (**b**) of *o*-Nb_2_O_5_ hollow microspheres, *t*-NbO_2_@carbon core-shell microspheres and *o*-Nb_2_O_5_@carbon core-shell microspheres.

The electrochemical behaviors of NbO_2_ and Nb_2_O_5_ are further studied by galvanostatic charge-discharge test. A comparison for all samples at a current density of 1 A g^−1^ during the initial discharge curve is shown in Fig. 5a. In the potential range of 1.0–2.0 V, all the discharge potential vary linearly with time, showing a good capacitive behavior as expected for a pseudocapacitive process. The shallower slope observed for *o*-Nb_2_O_5_@carbon is consistent with its higher capacitance. The rate capabilities of all samples from 1 to 50 A g^−1^ are compared in Fig. 5b. At a low current density of 1 A g^−1^ (5 C), *t*-NbO_2_@carbon has a specific capacitance of 377.1 F g^−1^ corresponding to 0.59 moL Li^+^ inserted in *t*-NbO_2_ (Li_0.59_NbO_2_), lower than that of *o*-Nb_2_O_5_ hollow (488.2 F g^−1^, Li_1.62_Nb_2_O_5_) and *o*-Nb_2_O_5_@carbon (502.1 F g^−1^, Li_1.66_Nb_2_O_5_). However, the capacity retention of *t*-NbO_2_ is much higher than those of *o*-Nb_2_O_5_ at high current densities. It still retains 245.8 F g^−1^ at 50 A g^−1^ (250 C), comparing to 126.1 F g^−1^ and 213.4 F g^−1^ for *o*-Nb_2_O_5_ hollow and *o*-Nb_2_O_5_@carbon, respectively. Such a high rate performance has rarely been reported for other pseudocapacitive metal oxides.

The ultrafast kinetics of Li^+^ intercalation in *t*-NbO_2_@carbon core-shell microspheres is particularly intriguing. However, it is not clear whether the observed behavior is unique to the nanostructure or the fundamental charge storage properties of *t*-NbO_2_ or the presence of impurity Nb_2_O_5_ in *t*-NbO_2_@carbon. To figure it out, commercial NbO_2_ and Nb_2_O_5_ powders (a high-purity of 99.9% for NbO_2_ and 99.99% for Nb_2_O_5_) were directly used as electrode materials. The typical SEM images are shown in Figure S8. The NbO_2_ powders consist of aggregated nanoparticles with average size of 20 μm, while Nb_2_O_5_ powders consist of scattered nanoparticles about 100 nm with slight aggregation, both of which are much larger than these prepared samples. The structure of commercial NbO_2_ and Nb_2_O_5_ powders has been compared by XRD, Raman and XPS results in Figure S9. The NbO_2_ and Nb_2_O_5_ exhibit the pure tetragonal and orthorhombic phase, respectively, which have the same crystal structure with our prepared samples.

**Figure 6:**
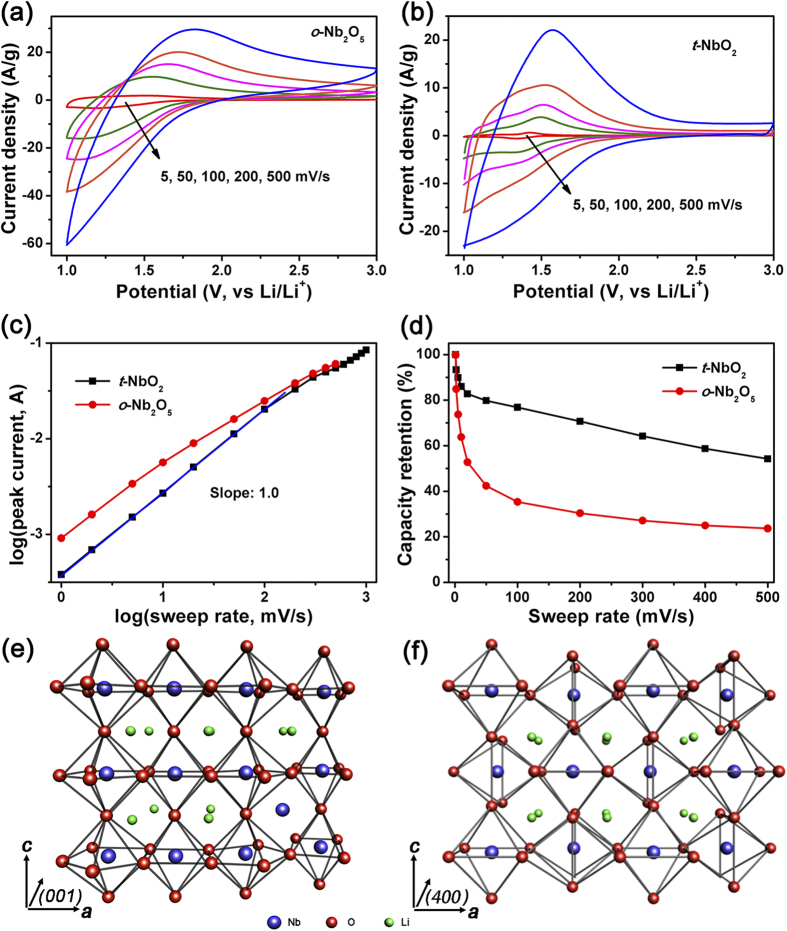
CV curves of commercial *o*-Nb_2_O_5_ powders (**a**) and *t*-NbO_2_ powders (**b**); the *b*-value of cathodic current peak (**c**); the capacity retention versus sweep rate (**d**); lithiated crystal structural schemes of *o*-Nb_2_O_5_ (**e**) and *t*-NbO_2_ (**f**).

The CV curves of commercial powders in sweep rate range from 5 to 500 mV s^−1^ are compared in Fig. 6a,b. These measurement conditions correspond to a charge or discharge time between 400 and 4 seconds. Moreover, comparing the electrochemical performance of the prepared nanoarchitectured Nb_2_O_5_ microspheres and commercial Nb_2_O_5_ powders in Figure S10, *o*-Nb_2_O_5_ hollow and o-Nb_2_O_5_@carbon exhibit the significantly higher specific capacitance and better rate capability than commercial *o*-Nb_2_O_5_ powders. This should be due to the improved electrochemical utilization of nanoarchitectured Nb_2_O_5_ hollow and *o*-Nb_2_O_5_@carbon with shorter Li^+^ diffusion path and more active sites.

The kinetics characterization is used to determine the charge storage behavior, whether it arises capacitive or diffusion-controlled processes. In Fig. 6c, the *b*-value of cathodic peak for commercial *t*-NbO_2_ is 1 in a wide sweep rate range (1 to 200 mV s^−1^), similar to what is observed with *t*-NbO_2_@carbon core-shell microspheres. The sweep-rate dependence of the capacity retention indicates that *t*-NbO_2_ electrode retains 54.3% of its maximum capacitance, much higher than 23.7% for *o*-Nb_2_O_5_ electrode at 500 mV s^−1^. These results verify that the ultrafast Li^+^ intercalation into *t*-NbO_2_ is an intrinsically capacitive process, and does not depend on the nanostructure of *t*-NbO_2_.

## Discussion

The nature of intercalation pseudocapacitive behavior of *o*-Nb_2_O_5_ is still a matter of debate, as the Li^+^ should meet a substantial resistance to insert into the densely packed crystal structure. Simon *et al.* proposed that intercalation pseudocapacitance observed with Nb_2_O_5_ is an intrinsic feature, arising from fast Li^+^ transport within the crystal structure^18^. Ganesh *et al.* concluded that it was due to the unique open channels of NbO_x_ sheets (similar to nano-porous structure) that reduce the energy barrier and facilitate the local charge transfer between lithium and oxygen structures^44^. In this work, we find that not only Nb_2_O_5_, but also NbO_2_ exhibits faster intercalation pseudocapacitive response. Apparently, these two materials have different structural and chemical similarities. As illustrated in Fig. 6e, the unit cell of *o*-Nb_2_O_5_ has sheets of edge- or corner-sharing distorted octahedral of [NbO_6_] and decahedron of [NbO_7_] lying parallel to (001) direction with 5% of the Nb^5+^ ions randomly located in 9-coordinate sites between (001) polyhedral planes. The rest of the empty octahedral sites provide natural tunnels for Li^+^ transport throughout the *ab* plane. While, the structure of *t*-NbO_2_ in Fig. 6f is only composed of corner- or edge-sharing octahedral of [NbO_6_]^18^,^44^,^45^. Both of *t*-NbO_2_ and *o*-Nb_2_O_5_ have such open channels of NbO_x_ sheets, thus giving arise to the possibility of pseudocapacitive mechanism. But, over a long-range, *t*-NbO_2_ is more structurally ordered than *o*-Nb_2_O_5_, which consists of distorted [NbO_6_] octahedral and [NbO_7_] decahedron. Thus, this may be one possible reason for the better kinetics of the former material as the intercalation material.

On the other hand, Li^+^ insertion into the structure dramatically increases the electronic conductivity of Nb_2_O_5_. Orel *et al.* showed that the conductivity of chemically-lithiated Nb_2_O_5_ was four orders of magnitude higher than that of Nb_2_O_5_^46^. Moreover, for the chemically delithiated LiNbO_2_, the Li_x_NbO_2_ (x < 1) is highly conductive and even becomes a superconductor at a transition temperature (T_c_) of ~5 K^47^,^48^. The stoichiometric compound LiNbO_2_ is a semi-conductive or semi-metallic behavior. However, the lithium-deficient Li_x_NbO_2_ (x < 1) is a superconductor with the superstructure derived from the ordering of the lithium vacancies in consecutive layers of [LiO_6_] octahedral. In this work, the NbO_2_ is converted into the Li_x_NbO_2_ (x ~ 0.59) during electrochemical Li^+^ intercalation process, which may undergo an insulator-to-metal transition (from NbO_2_ to Li_x_NbO_2_).

Furthermore, the EIS results of before and after lithiation in *t*-NbO_2_@carbon and *o*-Nb_2_O_5_@carbon were shown in Figure S11. Simulations indicate that the faradaic charge transfer resistance (R_pseudo_) decreases obviously on lithiation from 3.9 to 3.2 ohms for *t*-NbO_2_, and 5.4 to 3.9 ohms for *o*-Nb_2_O_5_, which means the lithiated compounds with the enhanced electronic conductivity. The phase angle of the slope line in low frequency increases slightly after lithiating to 1.0 V for NbO_2_ and Nb_2_O_5_, indicating a better Li^+^ diffusion in the lithiated compounds. Thus, we proposed that during electrochemically intercalation process, the NbO_2_ was changed into Li_x_NbO_2_, which may also go through a semiconductor to conductive transition. The enhanced electronic conductivity is in favour of electrostatic adsorption of Li^+^ onto the surface of lithiated Li_x_NbO_2_ nanocrystals and thus promoting the fast ion diffusion in the bulk. Although a detailed reaction mechanism has not been reached, we speculate that the high-rate intercalation behavior of this system results from not only a structure with two-dimensional transport pathways and little structural change on intercalation, but also the formation of conductive lithiated compounds with no limitations of the surface adsorption and surface transfer. However, whether as-obtained lithiated Li_x_NbO_2_ exhibits high conductivity, it should need further resultant conductivity test to check this hypothesis.

In summary, we successfully construct a series of nanoarchitectured intercalation materials including *o*-Nb_2_O_5_ hollow microspheres, *o*-Nb_2_O_5_@carbon and *t*-NbO_2_@carbon core-shell microspheres for ultrahigh-rate Li^+^ intercalation pseudocapacitors. In these core-shell nanostructures, the conductive carbon core could mitigate the low electronic conductivity of nanocrytals. On the other hand, the 3D urchin-like shell structure assembled by numerous nanorods could increase the utilization degree of the nanocrystals and improve the electrode kinetics. Thus, *o*-Nb_2_O_5_@carbon core-shell microspheres show higher electrochemical utilization and faster rate handling properties compared with *o*-Nb_2_O_5_ hollow microspheres. And more importantly, we find for the first time, the *t*-NbO_2_ exhibits much better Li^+^ intercalation kinetics than the *o*-Nb_2_O_5_, even no diffusion limitations for charging times as fast as 5.9 s (250 C rate). The more ordered crystal structure of *t*-NbO_2_ and enhanced conductivity of Li_x_NbO_2_ might be the reasons for faster kinetics of *t*-NbO_2_. We speculate that the origin of high-rate pseudocapacitance in Nb_2_O_5_ and NbO_2_ are more likely due to the lithiated compounds with dramatically conductivity instead of their crystal structure. A wide range of possibilities to modify the crystalline and physical properties of Nb-O derived structures would probably lead to materials with both high energy density and high power density, which will confuse the distinction between supercapacitors and batteries.

## Methods

### Synthesis

The *o*-Nb_2_O_5_ hollow microspheres, *o*-Nb_2_O_5_@carbon and *t*-NbO_2_@carbon core-shell microspheres were fabricated through a facile one-pot hydrothermal method followed with different post-treatments. In a typical process, 1.368 g of resorcinol (R) 2.016 g of formaldehyde (F, 37%) and 1.824g of ammonium niobate oxalate hydrate (C_4_H_4_NNbO_9_•*x*H_2_O, 99.9%, Sigma-Aldrich) were mixed in 70 mL DI-water at room temperature. After stirring for 4 h at 40 ^o^C, the solution was transferred to a 90 mL Teflon-lined autoclave and heated at 180 ^o^C for 24 h. The resulting precipitates were collected, filtered, washed with DI-water several times, then dried at 80 ^o^C for 24 h to obtain Nb_2_O_5_@ploymer core-shell microspheres. The obtained Nb_2_O_5_@ploymer core-shell microspheres were treated with the followed post-processing: (Step I) calcination in air flow at 600 ^o^C for 2 h with heating rate 2 ^o^C min^−1^ to obtain *o*-Nb_2_O_5_ hollow microspheres; (Step II) carbonization at 800 ^o^C for 2 h with heating rate 3 ^o^C min^−1^ under N_2_ flow to obtain *t*-NbO_2_@carbon core-shell microspheres; (Step III) a mild oxidation of *t*-NbO_2_@carbon core-shell microspheres in air flow at 300 ^o^C for 2h, and *t*-NbO_2_ phase could transfer into *o*-Nb_2_O_5_ phase. The synthesis of pure carbon microspheres is similar to that for composite microspheres but without adding the C_4_H_4_NNbO_9_•*x*H_2_O. The obtained RF polymeric microspheres were carbonized at 800 ^o^C for 2 h with heating rate 3 ^o^C min^−1^ under N_2_ flow.

### Characterization

The crystal structure of all the samples was identified by a powder X-ray diffraction (XRD) patterns with a RigakuD/max 2550 diffractometer operating at 40 kV and 20 mA using Cu Kα radiation (λ = 1.5406 Å). The Raman spectra was recorded at room temperature on a Spex 1403 Raman spectrometer with an argon ion laser at an excitation wavelength of 514.5 nm. X-ray photoelectron spectroscopy (XPS) analysis was performed using a VG Multilab 2000 with Al Kα as the X-ray source. The surface morphology and microstructure were observed on field emission scanning electron microscopy (SEM, FEI-300) and transmission electron microscopy (TEM, JEOL, 2100F). Nitrogen adsorption/desorption isotherms were measured at 77 K with a Quadrasorb SI analyser. Before the measurements, all samples were degassed under vacuum at 453 K for 12 h. Brunauer-Emmett-Teller (BET) method was utilized to calculate the specific surface areas. The total pore volume was calculated using a single point at a relative pressure of 0.985. The pore size distributions were derived from the desorption branch using Barrett-Joyner-Halenda (BJH) model. Thermogravimetric analysis (TA Instrument Q600 Analyser) was carried out in air flow rate of 100 mL min^−1^ from room temperature to 800 ^o^C at a rate of 10 ^o^C min^−1^.

### Electrochemical tests

The electrode slurry was prepared by mixing the as-prepared material, carbon black (Timical super C65) and polyvinylidene fluoride (PVDF) binder in a 8:1:1 weight ratio in N-methyl-2-pyrrolidinone (NMP). Then the slurry was uniformly casted onto Cu foil, dried in a 100 ^o^C vacuum oven overnight, and punched into electrodes with a diameter of 12 mm and a thickness of 50 μm (not include Cu foil). Electrochemical tests were performed in 3-electrode system with the obtained samples as working electrode, overcapacitive activated carbons and lithium foil as the counter and reference electrode, respectively. 1 M LiPF_6_ in EC/DMC/EMC (V/V, 1:1:1) was employed as the electrolyte, the separator was a microporous membrane (Celgard 2400). Cyclic voltammetry (CV) was conducted on a PCI-4/300 potentiostat (Gamry, USA) and galvanostatic charge-discharge (GCD) tests was conducted on the Arbin BT2000 system. All the soft cells were assembled in an argon filled glove box at 25 ^o^C and each cell need to be injected 0.5 mL electrolyte. Cyclic voltammetry (CV) and galvanostatic charge-discharge (GCD) were tested in the potential range between 1.0 V and 3.0 V at room temperature. The weight of active materials (Nb_2_O_5_ or NbO_2_) in electrode was used to calculate the gravimetrically normalized current and capacitance.

## Additional Information

**How to cite this article**: Kong, L. *et al.* Nanoarchitectured Nb_2_O_5_ hollow, Nb_2_O_5_@carbon and NbO_2_@carbon Core-Shell Microspheres for Ultrahigh-Rate Intercalation Pseudocapacitors. *Sci. Rep.* **6**, 21177; doi: 10.1038/srep21177 (2016).

## Supplementary Material

Supplementary Information
