# Supplementary material for: Nanoarchitectured Nb2O5 hollow, Nb2O5@carbon and NbO2@carbon Core-Shell Microspheres for Ultrahigh-Rate Intercalation Pseudocapacitors
Source: Sci Rep. 2016 Feb 16;6:21177. doi: 10.1038/srep21177 (PMC4754730; doi:10.1038/srep21177)
Supplement: Supplementary Information [file srep21177-s1.pdf]

Supplementary Information

Nanoarchitectured Nb_2O_5 hollow, Nb_2O_5@carbon and NbO_2@carbon Core-Shell Microspheres for Ultrahigh-Rate Intercalation Pseudocapacitors

Lingping Kong,[a] Chuanfang Zhang,[a] Jitong Wang,[a] Wenming Qiao,[a,b] Licheng Ling [a,b] and Donghui Long[a,b]*

[a.] *State Key Laboratory of Chemical Engineering, East China University of Science and Technology, Shanghai 200237, China.*

[b.] *Key Laboratory of Specially Functional Polymeric Materials and Related Technology, East China University of Science and Technology, Shanghai 200237, China.*

[*] Corresponding author: Donghui Long, E-mail: longdh@mail.ecust.edu.cn.

Tel: +86 21 64252924. Fax: +86 21 64252914.

This PDF file includes:

Figure S1 to S11

Table S1

Content:

Figure S1. XRD pattern (a) and SEM image (b) of Nb_2O_5@polymer core-shell microspheres.

Figure S2. SEM images of *t*-NbO_2@carbon core-shell microspheres with different hydrothermal time: 1 h (a-b); 3 h (c-d); 9 h (e-f).

Figure S3. TG analysis of *o*-Nb_2O_5 hollow microspheres, *t*-NbO_2@carbon core-shell microspheres and *o*-Nb_2O_5@carbon core-shell microspheres in air flow.

Figure S4. N_2 adsorption-desorption isotherms (a) and BJH pore size distributions (b) of *o*-Nb_2O_5 hollow microspheres, *t*-NbO_2@carbon core-shell microspheres and *o*-Nb_2O_5@carbon core-shell microspheres.

Figure S5. SEM images of *o*-Nb_2O_5 hollow microspheres, *t*-NbO_2@carbon core-shell microspheres and *o*-Nb_2O_5@carbon core-shell microspheres.

Figure S6. SEM image of carbon microspheres (a), CV curves of carbon microspheres and *o*-Nb_2O_5@carbon core-shell microspheres at 10 mV s^{-1} in $LiPF_6$ electrolyte (b).

Figure S7. CV curves of *o*-Nb_2O_5 hollow, *o*-Nb_2O_5@carbon and *t*-NbO_2@carbon microspheres (a), and commercial *o*-Nb_2O_5 and *t*-NbO_2 powders (b) at 1 mV s^{-1}.

Figure S8. SEM images of commercial *t*-NbO_2 powders (a-b) and commercial *o*-Nb_2O_5 powders (c-d) without any treatment.

Figure S9. XRD pattern (a), Raman spectra (b) and high-resolution Nb3d XPS spectrum (c) of commercial *t*-NbO_2 and *o*-Nb_2O_5 powders without any treatment.

Figure S10. Specific capacitance versus sweep rate of *o*-Nb_2O_5 hollow microspheres, *o*-Nb_2O_5@carbon core-shell microspheres and commercial *o*-Nb_2O_5 powders.

Figure S11. Electrochemical impedance spectroscopy of *t*-NbO_2@carbon (a) and *o*-Nb_2O_5@carbon (b) before any lithiation at open-circuit and after lithiating to 1.0 V. The equivalent circuit model (c).

Table S1. Porosity parameters of *o*-Nb_2O_5 hollow microspheres, *t*-NbO_2@carbon core-shell microspheres and *o*-Nb_2O_5@carbon core-shell microspheres.

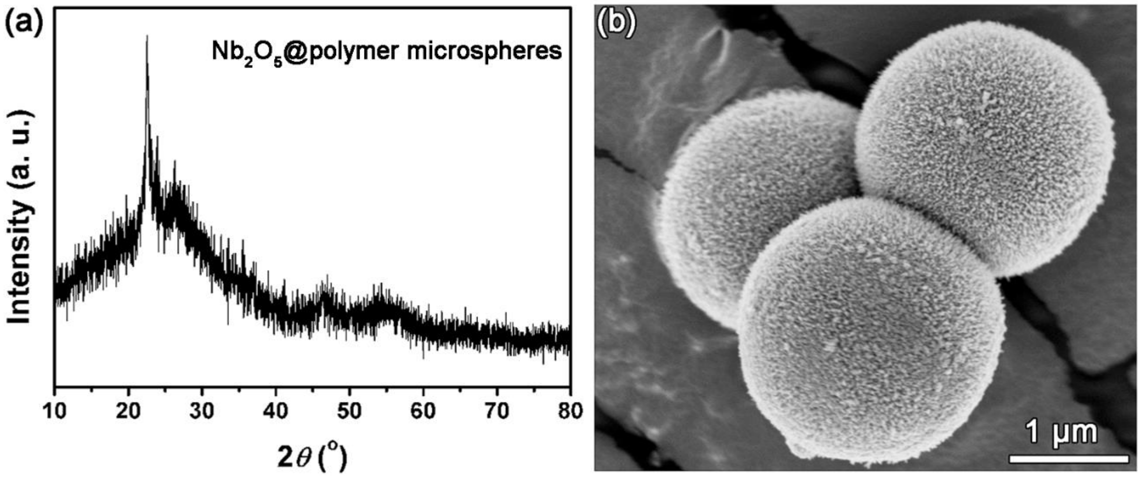

Figure S1. XRD pattern (a) and SEM image (b) of Nb_2O_5@polymer core-shell microspheres.

After hydrothermal process, Nb_2O_5@polymer core-shell microspheres were obtained. The amorphous phase Nb_2O_5 could be confirmed by XRD with the broad diffraction peaks. The morphologies of Nb_2O_5@polymer core-shell microspheres were observed by SEM. The spherical particles have their uniform diameter about 2-3 μm and their surface are not smooth but with urchin-like shell assembled by numerous nanorods protruding radially from the center.

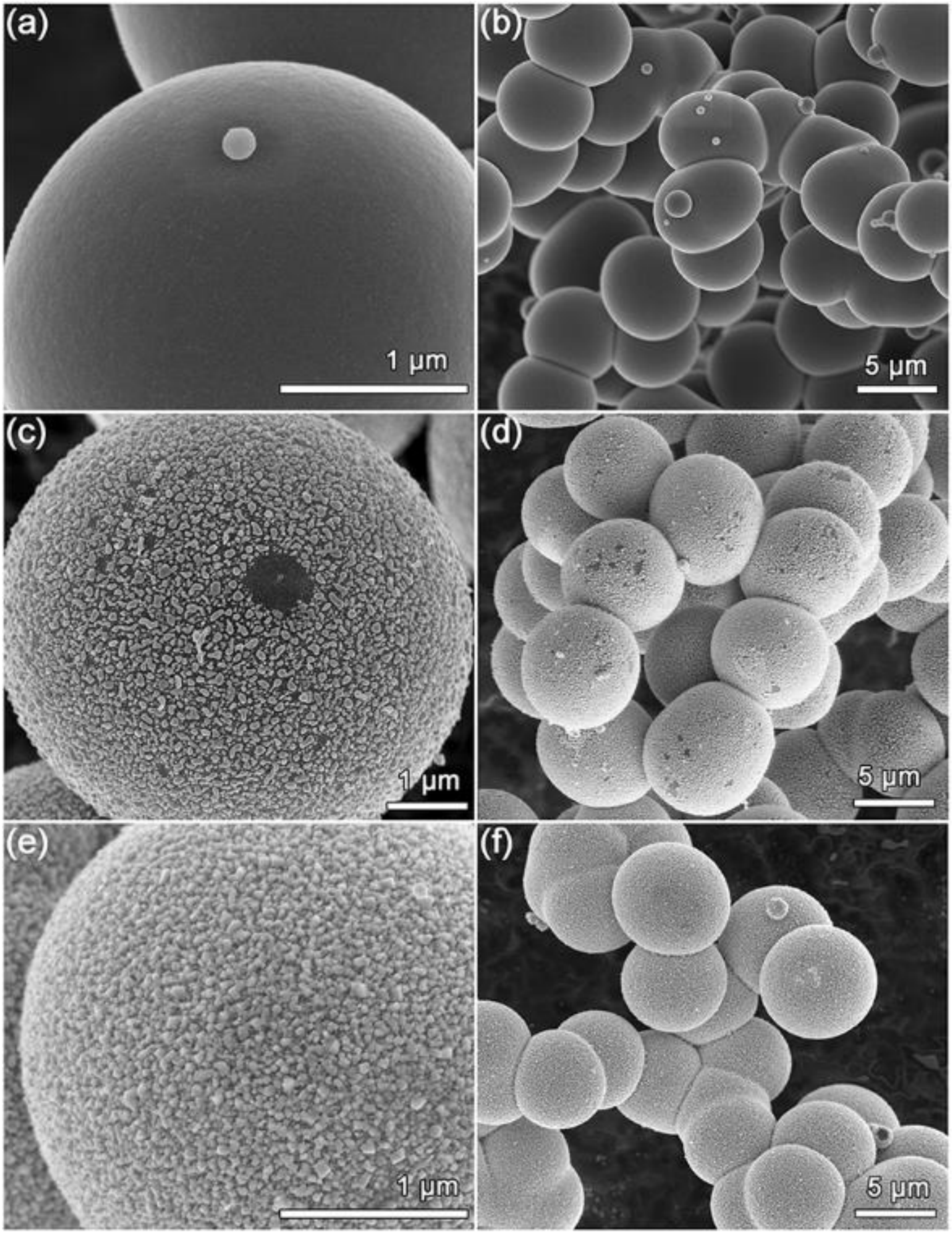

Figure S2. SEM images of *t*-NbO_2@carbon core-shell microspheres with different hydrothermal time: 1 h (a-b); 3 h (c-d); 9 h (e-f).

The formation mechanism of Nb_2O_5@polymer core-shell microspheres could be verified by a hydrothermal time-dependent experiment. After hydrothermal time for 1h, the carbon microspheres have the smooth surface. With the hydrothermal time increasing, the surface of microspheres are not smooth, but coating with more and more nanoparticles until formation thin shell of Nb_2O_5. Therefore, the fabrication process is accomplished by the "couple synthesis" approach, which should involve the fast formation of RF polymeric microspheres *in situ*, following by the hetero-nucleation and growth of Nb_2O_5 nanoparticles on the RF colloidal microsphere surfaces.

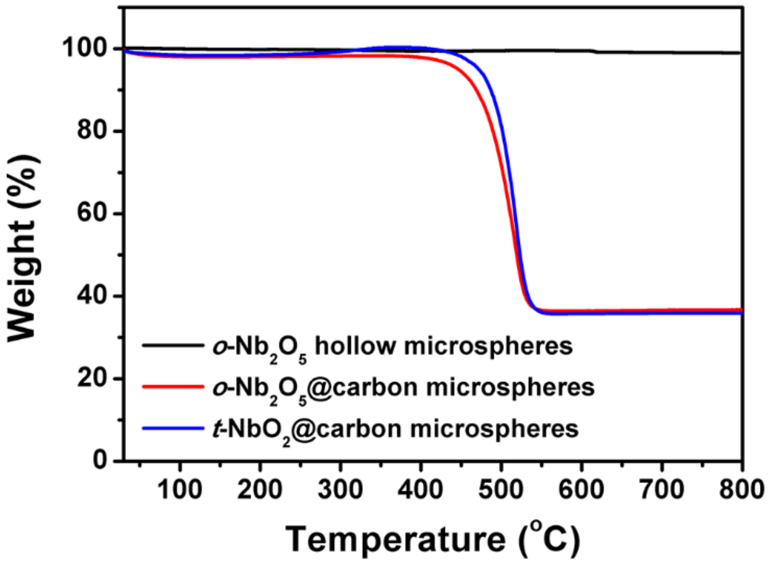

Figure S3. TG analysis of *o*-Nb_2O_5 hollow microspheres, *t*-NbO_2@carbon core-shell microspheres and *o*-Nb_2O_5@carbon core-shell microspheres in air flow.

The TG curve of *o*-Nb_2O_5 hollow microspheres is flat with a slight weight loss during heat treatment. The *o*-Nb_2O_5@carbon core-shell microspheres has a weight lost at around 420 ^oC and the weight contents of Nb_2O_5 in the composite microspheres is 34.0%. While the *t*-NbO_2@carbon core-shell microspheres has slight weight increase at 300 ^oC, then weight lost at about 410 ^oC, that's because the NbO_2 was oxidized to Nb_2O_5 under oxygen atmosphere. The weight contents of NbO_2 in the composite microspheres is confirmed to 33.3% by calculation.

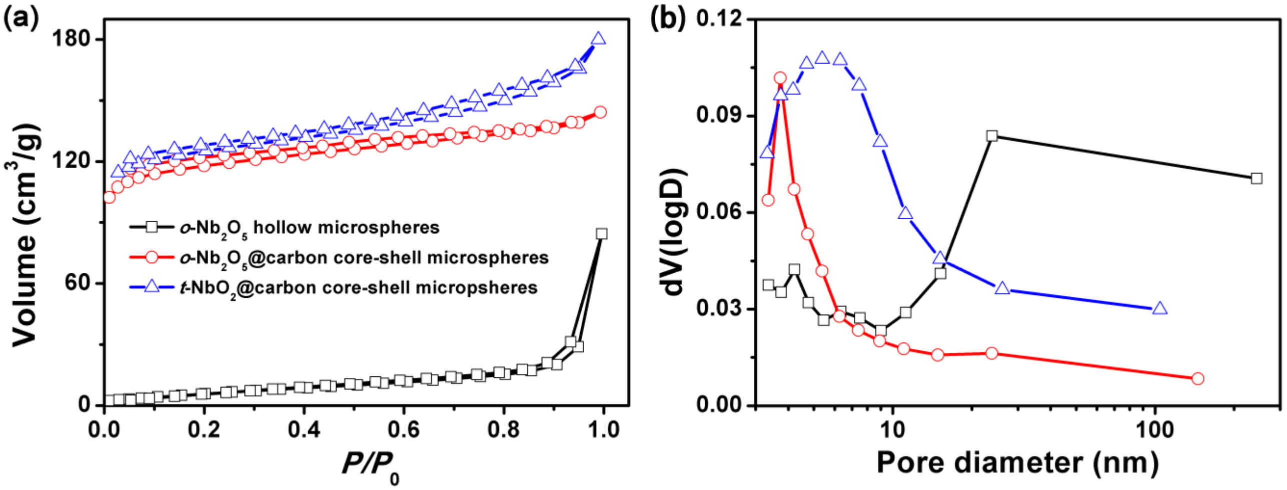

Figure S4. N_2 adsorption-desorption isotherms (a) and BJH pore size distributions (b) of *o*-Nb_2O_5 hollow microspheres, *t*-NbO_2@carbon core-shell microspheres and *o*-Nb_2O_5@carbon core-shell microspheres.

The BET surface area of *o*-Nb_2O_5 hollow microspheres is 26 m^2/g. After compositing with carbon core, the BET surface areas of *t*-NbO_2@carbon and *o*-Nb_2O_5@carbon core-shell microspheres increase to 473 and 456 m^2/g respectively. The increased surface areas are apparently due to the contribution of microporous carbon core.

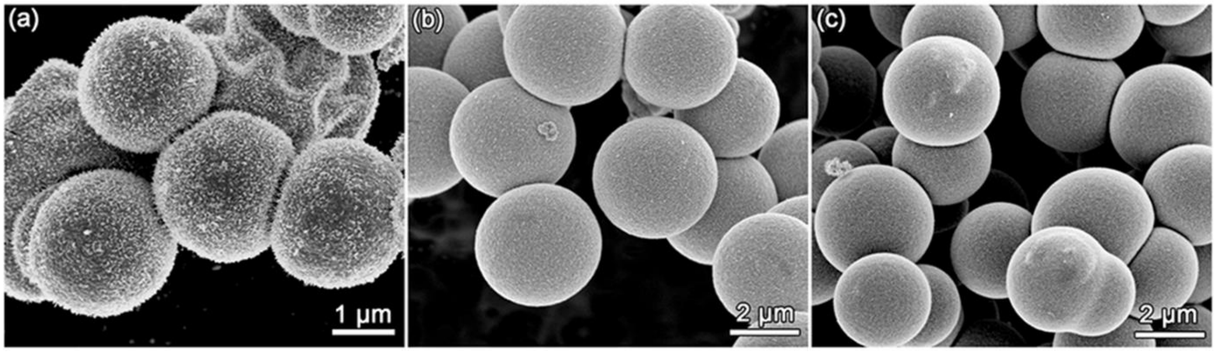

Figure S5. SEM images of *o*-Nb_2O_5 hollow microspheres, *t*-NbO_2@carbon core-shell microspheres and *o*-Nb_2O_5@carbon core-shell microspheres.

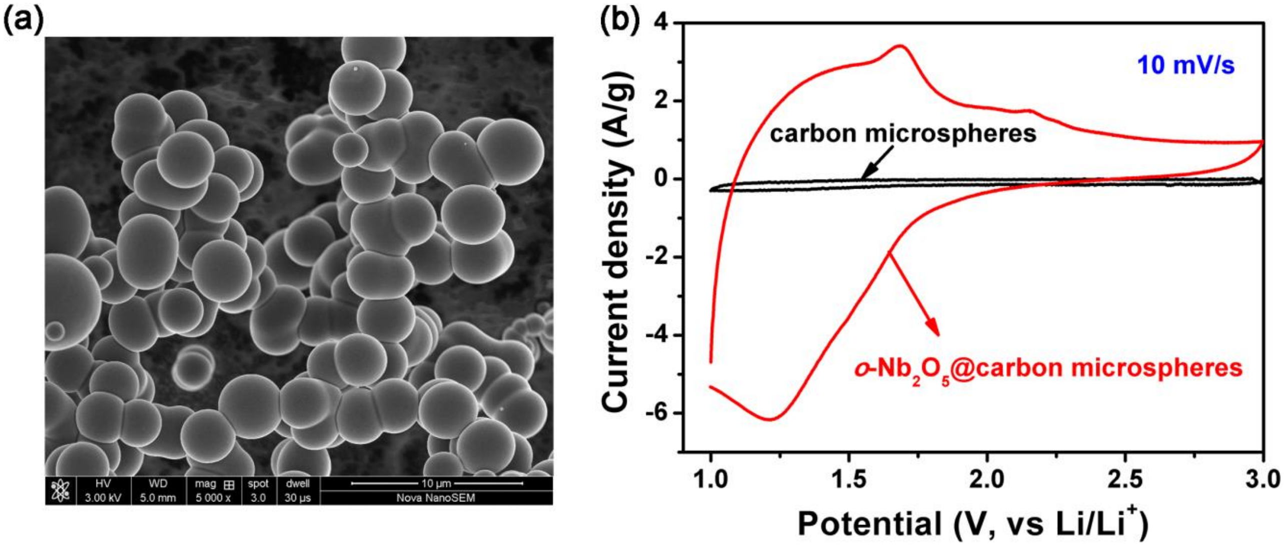

Figure S6. SEM image of carbon microspheres (a), CV curves of carbon microspheres and *o*-Nb_2O_5@carbon core-shell microspheres at 10 mV s^{-1} in $LiPF_6$ electrolyte (b).

The synthesis of pure carbon microspheres was similar to that for composite microspheres but without adding the $C_4H_4NNbO_9{\bullet}xH_2O$. Comparing the CV curves of pure carbon microspheres and *o*-Nb_2O_5@carbon core-shell microspheres in Figure S6 b, we found that the electric double-layer capacitance of carbon microspheres was negligible compare to the pseudo-capacitance of *o*-Nb_2O_5.

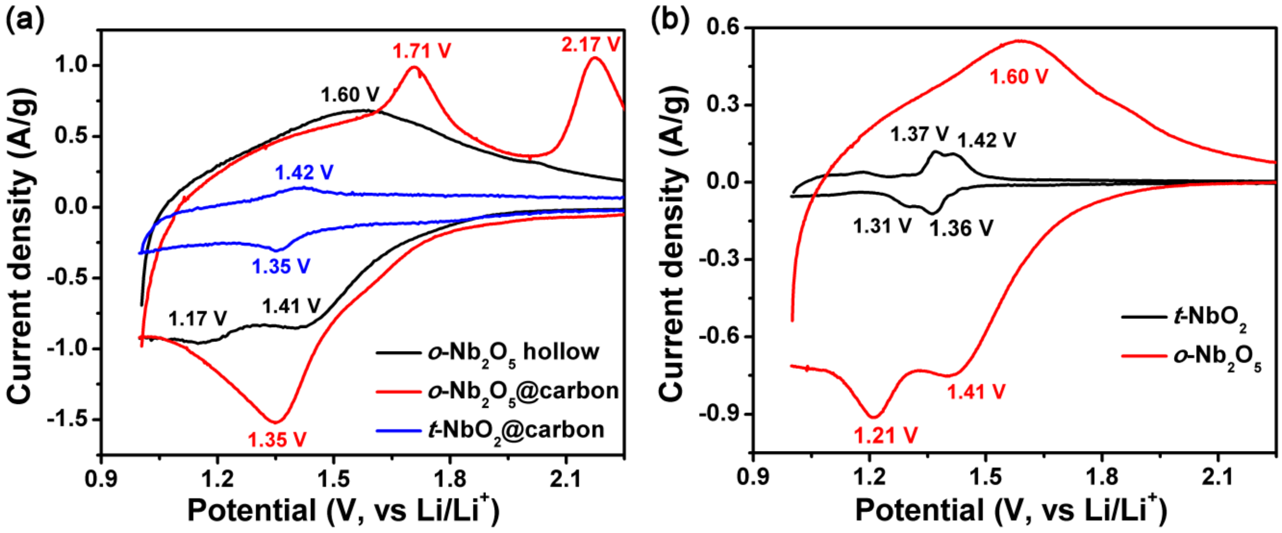

Figure S7. CV curves of *o*-Nb_2O_5 hollow, *o*-Nb_2O_5@carbon and *t*-NbO_2@carbon microspheres (a), and commercial *o*-Nb_2O_5 and *t*-NbO_2 powders (b) at 1 mV s^{-1}.

Nb^{4+} is in NbO_2 and Nb^{5+} is in Nb_2O_5, leading to different lithiated products (Li_xNbO_2 and $Li_xNb_2O_5$) during the discharging process. Apparently, the potential for Nb^{4+} to Nb^{3+} redox is different to the potential for Nb^{5+} to Nb^{4+} redox. The Li^+ insertion and extraction peaks of *t*-NbO_2@carbon are at around 1.35 V and 1.42 V, respectively. While, *o*-Nb_2O_5 hollow sample shows two insertion peaks at around 1.17 V and 1.41 V and one broad extraction peaks at 1.60 V. Moreover, the CV curves for the commercial *t*-NbO_2 and *o*-Nb_2O_5 powders also exhibit the different redox peaks of *t*-NbO_2 and *o*-Nb_2O_5.

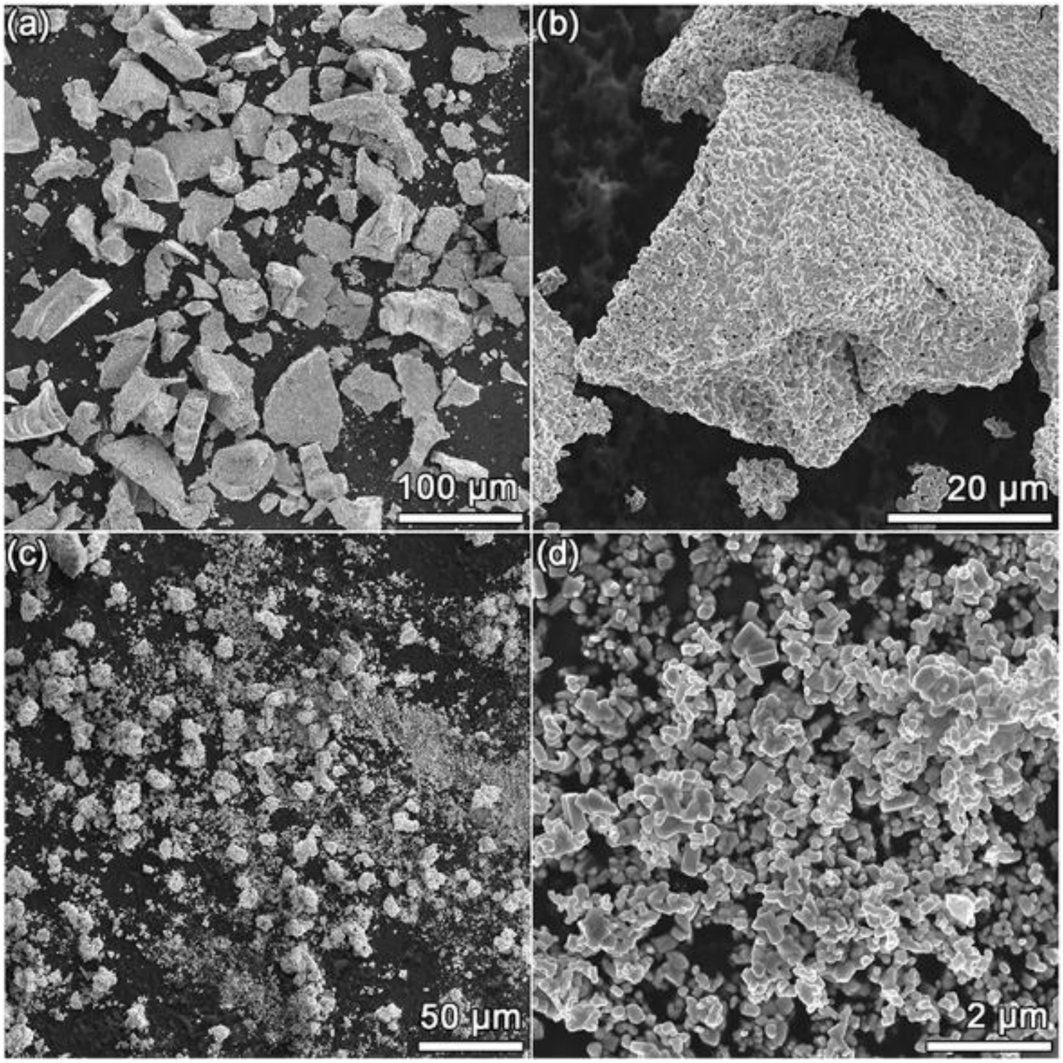

Figure S8. SEM images of commercial *t*-NbO_2 powders (a-b) and commercial *o*-Nb_2O_5 powders (c-d) without any treatment.

The morphology of these commercial powders are observed by SEM. Commercial *t*-NbO_2 powders are consisted of aggregated nanoparticles with average size of 20 μm and *o*-Nb_2O_5 powders are consisted of scattered nanoparticles about 100 nm with slight aggregation. Their sizes are much larger than these nanostructured rod crystals.

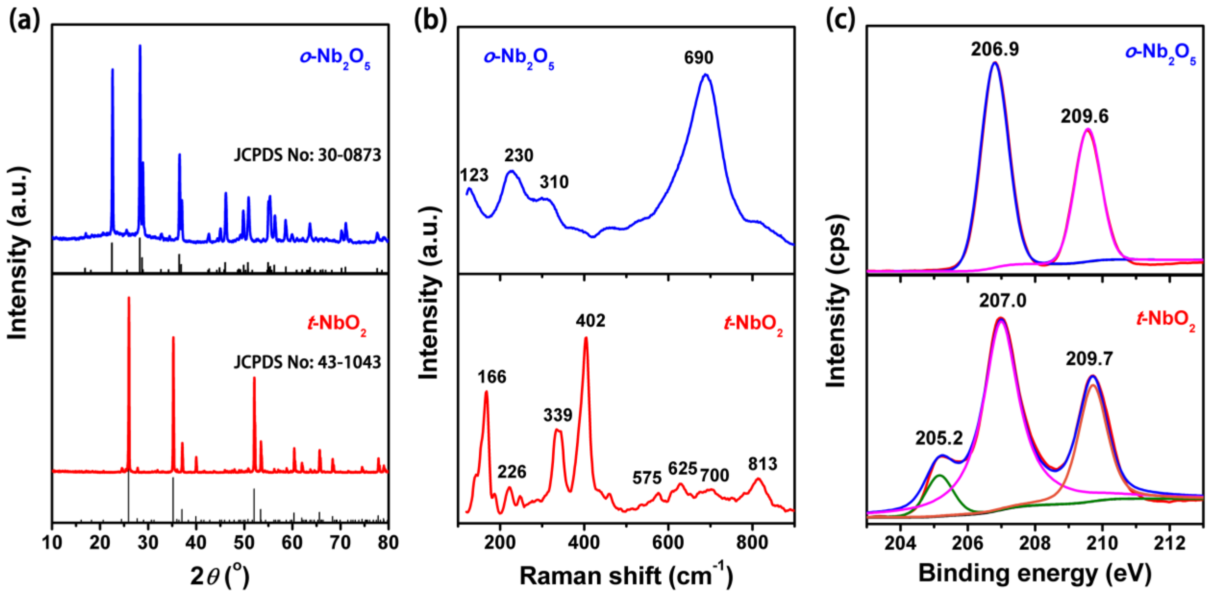

Figure S9. XRD pattern (a), Raman spectra (b) and high-resolution Nb3d XPS spectrum (c) of commercial *t*-NbO_2 and *o*-Nb_2O_5 powders without any treatment.

The Nb_2O_5 and NbO_2 reference materials were obtained in the form of a high-purity powder (99.9% for NbO_2 and 99.99% for Nb_2O_5, trace metal basis) from Sigma-Aldrich. The orthorhombic phase of commercial Nb_2O_5 can be well confirmed by its XRD pattern, which can be indexed to JCPDS No. 30-0873. Meanwhile, the specific Raman vibrational modes centered at 123 cm^{-1} (v_1), 230 cm^{-1} (v_2), 310 cm^{-1} (v_3) and 690 cm^{-1} (v_4) also confirm the pure orthorhombic phase. The high-resolution Nb3d XPS spectrum shows two narrow peaks at 206.9 eV and 209.6 eV, corresponding to the spin doublet $3d_{5/2}$ and $3d_{3/2}$, in good agreement with the binding energies of Nb_2O_5.

Commercial NbO_2 exhibits a high degree of crystallinity with tetragonal phase, because all the diffraction peaks can be indexed to JCPDS No. 43-1043. The good agreement observed between the theoretically and experimentally determined positions of the peaks confirms the crystal structure of the NbO_2 reference material and no impurities are apparent in the XRD data. Meanwhile, the peaks in Raman spectrum of commercial *t*-NbO_2 are good consistent with the result

of single crystalline NbO_2 nanowire reported by Lee et al. ("Single Crystalline NbO_2 Nanowire Synthesis by Chemical Vapor Transport Method" Bull. Korean Chem. Soc. 2012, 33, 839). In addition, electron spectra recorded for commercial *t*-NbO_2 shows two peaks (207.0 eV and 209.7 eV) with approximately same binding energies as observed for Nb_2O_5 and a shoulder peak at lower binding energy (205.2 eV) with smaller intensity. This Nb 4d spectrum of NbO_2 is broadly in agreement with that previous reported ("Photoemission and STM study of the electronic structure of Nb-doped TiO_2" D. Morris et al, Physical Review B 2000, 61, 13445-13457). In the case of NbO_2, the binding energies of the spin-orbit components associated with poorly screened final state coincide with those found for Nb_2O_5, so that the poorly screened state corresponds to a $4d^0$ final-state configuration. All these results confirm that the commercial NbO_2 is pure *t*-NbO_2 phase in bulk.

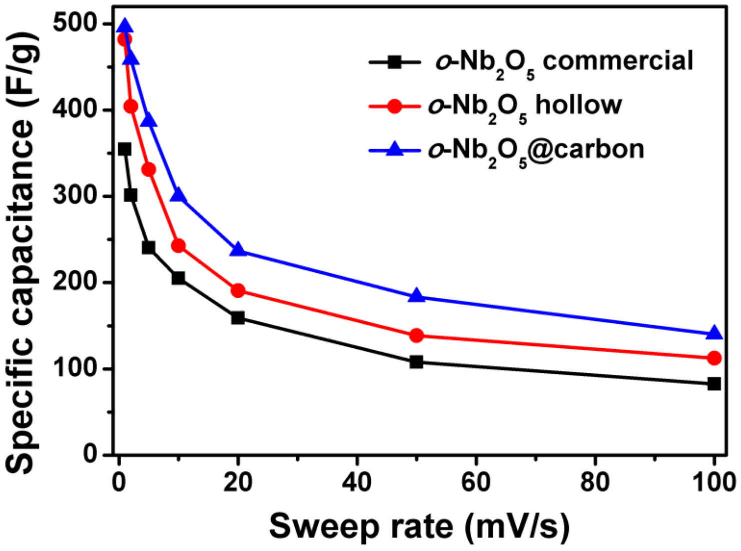

Figure S10. Specific capacitance versus sweep rate of *o*-Nb_2O_5 hollow microspheres, *o*-Nb_2O_5@carbon core-shell microspheres and commercial *o*-Nb_2O_5 powders.

The specific capacitance and rate capability of nanoarchitectured *o*-Nb_2O_5 hollow and *o*-Nb_2O_5@carbon microspheres are significantly higher than commercial Nb_2O_5 powders. This should be due to the improved electrochemical utilization of nanoarchitectured Nb_2O_5 hollow and *o*- Nb_2O_5@carbon microspheres with shorter Li^+ diffusion path and more active sites.

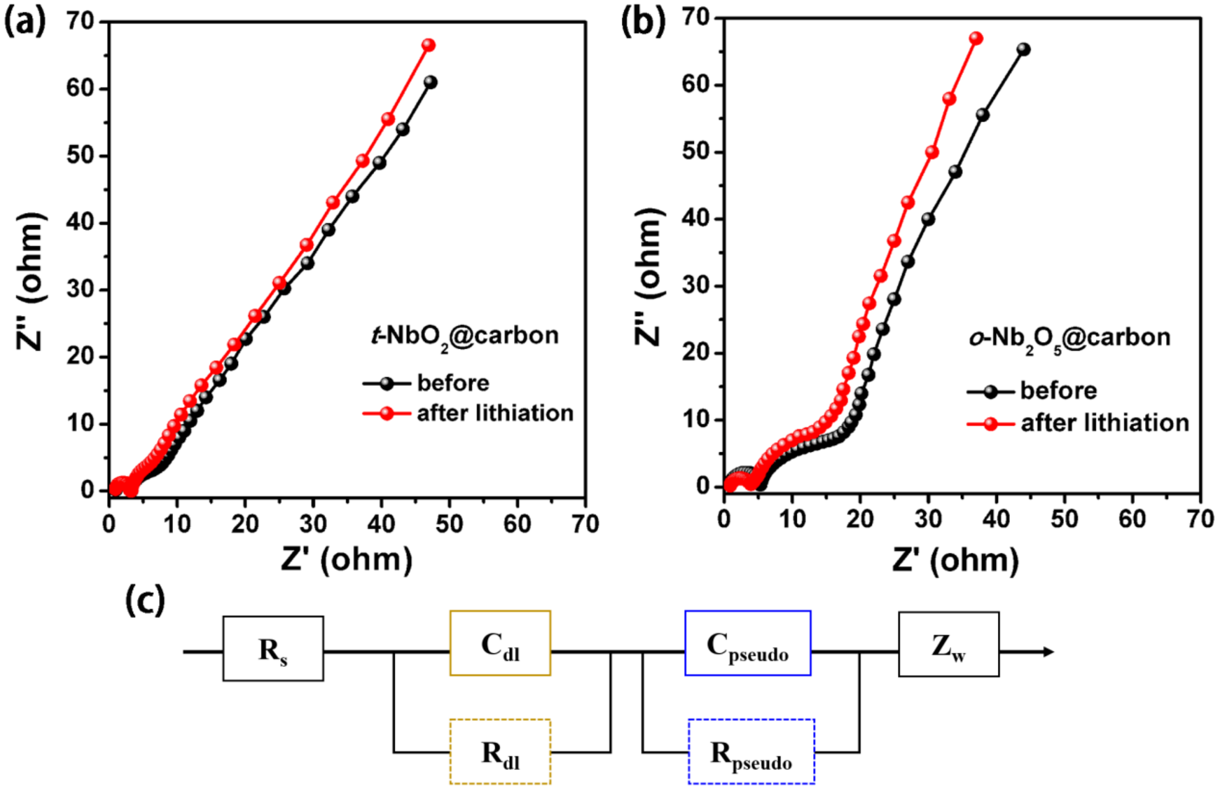

Figure S11. Electrochemical impedance spectroscopy of *t*-NbO_2@carbon (a) and *o*-Nb_2O_5@carbon (b) before any lithiation at open-circuit and after lithiating to 1.0 V. The equivalent circuit model (c).

As in Figure S11 a, the charge transfer resistance of lithiated Li_xNbO_2 is much lower than that of NbO_2, which may due to the Li^+ intercalation into NbO_2 improving the electronic conductivity. The similar results were also observed in $Li_xNb_2O_5$. The equivalent circuit model includes R_s (the resistance of electrolyte, microporous membrane, wire, etc.), R_{dl} (electrode/electrolyte interfaces), R_f (faradaic charge transfer resistance) and Z_w (Warburg resistance), as shown in Figure S11 c. Simulations indicate that R_{pseudo} decreases on lithiation from 3.9 to 3.2 ohms for NbO_2, and 5.4 to 3.9 ohms for Nb_2O_5. Moreover, deviation from a vertical line to phase angles of < 90° often occurs and can indicate pseudocapacitive behavior, which is often represented by a constant-phase element in the equivalent circuit:

$$Z_w = \frac{1}{B(j\omega)^n}$$

Here, Z is the impedance, B is a constant, and ω is the frequency. The phase angle of the slope line in low frequency increases slightly after lithiating to 1.0 V for NbO_2 and Nb_2O_5, indicating a better Li^+ diffusion in the lithiated compounds.

Table S1. Porosity parameters of *o*-Nb_2O_5 hollow microspheres, *t*-NbO_2@carbon core-shell microspheres and *o*-Nb_2O_5@carbon core-shell microspheres.

Sample	$^aS_{BET}$ / m^2 g^{-1}	^bV_T / cm^3 g^{-1}
o-Nb_2O_5 hollow microspheres	26	0.13
o-Nb_2O_5@carbon core-shell microspheres	456	0.23
t-NbO_2@carbon core-shell microspheres	473	0.28

[a] BET specific surface area; [b] total pore volume
